# Potentially avoidable hospitalisation for constipation in Victoria, Australia in 2010–11

**DOI:** 10.1186/1471-230X-14-125

**Published:** 2014-07-11

**Authors:** Humaira Ansari, Zahid Ansari, John M Hutson, Bridget R Southwell

**Affiliations:** 1 Surgical Research Group, Murdoch Childrens Research Institute, Flemington Road, Parkville 3052, Victoria, Australia; 2 School of Public Health and Preventive Medicine, Monash University, Clayton, Victoria, Australia; 3 Department of Paediatrics, University of Melbourne, Parkville 3052, Victoria, Australia; 4 Department of Urology, Royal Children’s Hospital, Parkville 3052, Victoria, Australia

## Abstract

**Background:**

When primary care of constipation fails, the patient may need emergency hospitalisation for disimpaction. This study aimed to provide population-based data on the number of unplanned admissions and the cost to the healthcare system for constipation in Victoria, Australia in financial year 2010–11.

**Methods:**

The Victorian Admitted Episodes Dataset was examined to find the number of emergency hospital separations coded as constipation (ICD-10-AM Code K390). An estimate of costs was determined from the number of weighted inlier equivalent separations (WIES) multiplied by the WEIS price, used by the Victorian Government for funding purposes.

**Results:**

There were 3978 emergency separations for constipation in Victoria in 2010–2011, 92% in public hospitals. Fifty-five percent were female and 38% > 75 years old. One third stayed overnight and 1/3 more than 1 day. The emergency bed day rate was 7.1 per 10,000 of population. The estimate of cost, based on WEIS, was approximately $8.3 million. Potential savings could be made by reducing the number of separations in 6 Local Government Areas (LGAs).

**Conclusions:**

This study shows that the burden (in number of admissions, emergency bed days and overall direct costs) in managing emergency admissions for constipation in Victoria, Australia, is very significant and likely to be similar in other developed countries. Improved primary healthcare and alternative ways to achieve faecal disimpaction without emergency admission could save the public health system a proportion of this $8.3 million.

## Background

Constipation, occurring as an idiopathic disorder or an adverse effect of lifestyle factors or medications, such as analgesics, antacids, antidepressants, antihistamines, anti-hypertensives, non-steroidal anti-inflammatory drugs, sedatives and opiates is common. There is a presumption that much of the hospitalisation that occurs for constipation should be able to be avoided. Most avoidable hospitalisations are emergency rather than elective admissions.

Potentially avoidable hospitalisations (Ambulatory Care Sensitive Conditions; ACSCs) are inpatient admissions which should not, in most cases, require hospital care. Proper community-based care for such conditions should delay the progression of the disease or prevent serious complications and thus prevent hospitalisation. Access to appropriate ambulatory care should prevent the onset of these types of illnesses or conditions, control acute episodes, or manage chronic diseases or conditions [1,2]. An emergency admission (separation) for an ACSC indicates poor overall quality-of-care even if the ACSC episode itself is managed well.

Admissions to hospital are an increasing pressure on health care budgets and avoidable admissions are an increasing focus of policy and program development. High ACSC admissions suggest poor coordination between different elements of a health care system, particularly primary and secondary care. In 2006, the United Kingdom Department of Health introduced a national target to reduce emergency bed-days by 5 percent by 2008 despite a projected increase in emergency admissions for ACSCs [3]. Nineteen conditions originally derived to measure access to primary care in the USA [1,2] were adapted as ACSC by [4] in Victoria. More recently, other conditions have been canvassed as ACSCs, including constipation, dyspepsia and other stomach disorders and migraine/acute headache [5,6].

The prevalence of constipation in the general population is estimated to be 17.1% in Europe and 15.3% in Oceania [7]. Constipation is widely viewed as a prevalent and benign condition not requiring medical advice given that short-term treatment (natural remedies or over-the-counter laxatives) is relatively straightforward. When poorly managed, constipation can give rise to more severe bowel complaints, including faecal impaction, that can lead to urinary and faecal incontinence [8]. Chronic constipation severely affects quality of life, which in children has been measured to be similar to patients with leukaemia [9].

This study aimed to provide population-based data on the number of admissions and level of funding associated with unplanned hospitalisations for constipation in hospitals in Victoria, Australia. More specifically, this study used administrative data to:

i. describe characteristics of patients and hospitals associated with emergency separations with a principal diagnosis of constipation in 2010–11;

ii. provide an estimate of the resources Victoria allocated to emergency separations for constipation in 2010–11 using the cross-sectional prevalence method;

iii. examine if there is a socio-economic gradient associated with emergencyseparations for constipation; and

iv. determine the proportion of growth in emergency separations for constipation between 2005–06 and 2010–11 arising from population growth, ageing and increased utilisation of hospital care.

## Methods

### Data sources

Each time a patient is admitted and discharged from hospital, it is counted as an episode-of-care, or separation. Data for this study were obtained from the Victorian Admitted Episodes Dataset (VAED). The VAED is an episode-based dataset containing data on all admitted patient activity in all public and private acute hospitals, including acute facilities in rehabilitation and extended care institutions and day-procedure centres, by financial year [10]. It includes information on patients’ demographic characteristics, selected institutional and residential geography-related variables, admission and discharge details and clinical data stored as International Classification of Diseases-10 Australian Modification (ICD-10-AM) codes in up to 40 diagnosis and procedure fields.

The VAED measures hospital activity for funding purposes but can also be used for epidemiological studies and health services research. The VAED is population-based, capturing data from all admitted patients for a defined geographic area (i.e. Victoria). There are standardised edit checks for unusual/missing values and audits because it provides a basis for funding hospital activity.

Emergency separations arise from admissions via Emergency Departments, general practitioners or self-referrals for acute illness. For this study, emergency hospital separations with a principal (first-listed) diagnosis of constipation (ICD-10-AM code K590) were defined as potentially avoidable hospitalisations due to the ACSC of constipation. The “principal” diagnosis is assigned after the patient’s condition has been investigated and is the reason for the patient being admitted. As such, it is the primary driver for the allocation to a diagnosis-related group (DRG).

### Statistical analysis

The data were analysed using Stata V-11. We calculated annual age- and sex-specific separation rates by dividing the number of separations attributed to the constipation ACSC by the population that year using 5-year age groups up to age 85 and people ≥ 85 years as a single age group. The population was calculated using the average of mid-year populations. All rates are expressed as: number of separations/10,000 population.

### Cost analysis

The expenditure associated with emergency separations was estimated by the casemix funding method that is applied annually to public hospitals in Victoria. Casemix systems allocate episodes of care to DRGs based on principal diagnosis (chief reason for patient’s episode-of-care in hospital), comorbidity, procedures, sex, age, mode of separation, length-of-stay and same-day status. The Victorian casemix system assigns a single cost weight (known as a “weighted inlier-equivalent separation” (WIES) value) to each episode-of-care that, reflects its cost relative to the cost of all episodes-of-care. The average cost of all episodes-of-care across all case-mix groups set as a reference point with a cost weight equal to one and the cost weight for each case-mix group (and each patients episode assigned to a case-mix group) is set relative to this reference point. The ‘standard’ or expected cost (that forms the basis for a hospital’s prospective payment for an episode of care) is equal to the cost weight multiplied by a scaling variable, the WIES price (which is set for each hospital payment group). The cost weight for each DRG is set annually based on an analysis of the reported episode-level costs incurred by Victorian hospitals incurred in treating patients.

Cost-weights do not take into account variations in hospital costs due to structural factors such as size, rural/metropolitan location, etc. The Department of Health uses different WIES prices for different types of hospitals to recognise differences in levels of specialisation, remoteness and economies-of-scale. For example, according to the Department of Health’s 2010-11 Policy and Funding Guidelines [11] the WIES prices ranged from $3,725-4,240.

Hospitals are funded upon the WIES they perform (up to a predetermined limit) times a unit price. This is considered to provide a reasonable basis for estimating the financial burden associated with providing secondary care from the Government perspective. In 2010–11 hospitals assigned diagnosis and procedure codes using the 7^th^ edition of the ICD-10-AM classification and the Department of Health funded hospitals based on WIES17 cost weights.

Because Australian private hospital separations are not subject to casemix funding, the funding for emergency separations for constipation was estimated separately for public hospital separations and for all separations. The lowest WIES price was applied to private separations to provide a population-based estimate of the financial burden of hospitalisations for constipation. As such, this study adopts a prevalence approach to estimating the costs associated with hospitalisation for constipation.

Analysis of separations, bed days and hospital-related costs by socio-economic status is based on the application of the Index of Relative Socio-Economic Disadvantage (IRSED) at the local government area (LGA) level. IRSED is the most commonly used area-level indicator of socio-economic status compiled by the Australian Bureau of Statistics from individual census variables aggregated at the local area level [12]. Each index consists of a set of area scores produced from principal components analysis of census-based socio-economic variables, including ethnic background, income, educational attainment, employment status and occupation summarised to have a mean of 1000 across all Census Collection Districts. IRSED defines disadvantage broadly in terms of access to material and social resources, and ability to participate in society. The higher the value of IRSED, the less disadvantaged the area is. For the 2006 census, IRSED values across Victorian LGAs ranged from 893.9 to 1,104.5. A population-based split of IRSED values was used to rank LGAs into 5 quintiles, from most disadvantaged (1st) to least disadvantaged (5th). The rate of hospitalisation was calculated by dividing the number emergency separations for constipation within a given IRSED quintile by the corresponding population in the same socioeconomic group.

Research was carried out in compliance with the Helsinki Declaration. Ethical approval was provided by the Human Research and Ethics Committee at the Royal Childrens Hospital (HREC 33509).

## Results

There were 3,978 emergency separations with a principal diagnosis of constipation in 2010–11, almost 1/3 more than the number of potentially preventable hospital separations for influenza and pneumonia and 3 times those for pelvic inflammatory disease and perforated/bleeding ulcer [13].

**Figure 1:**
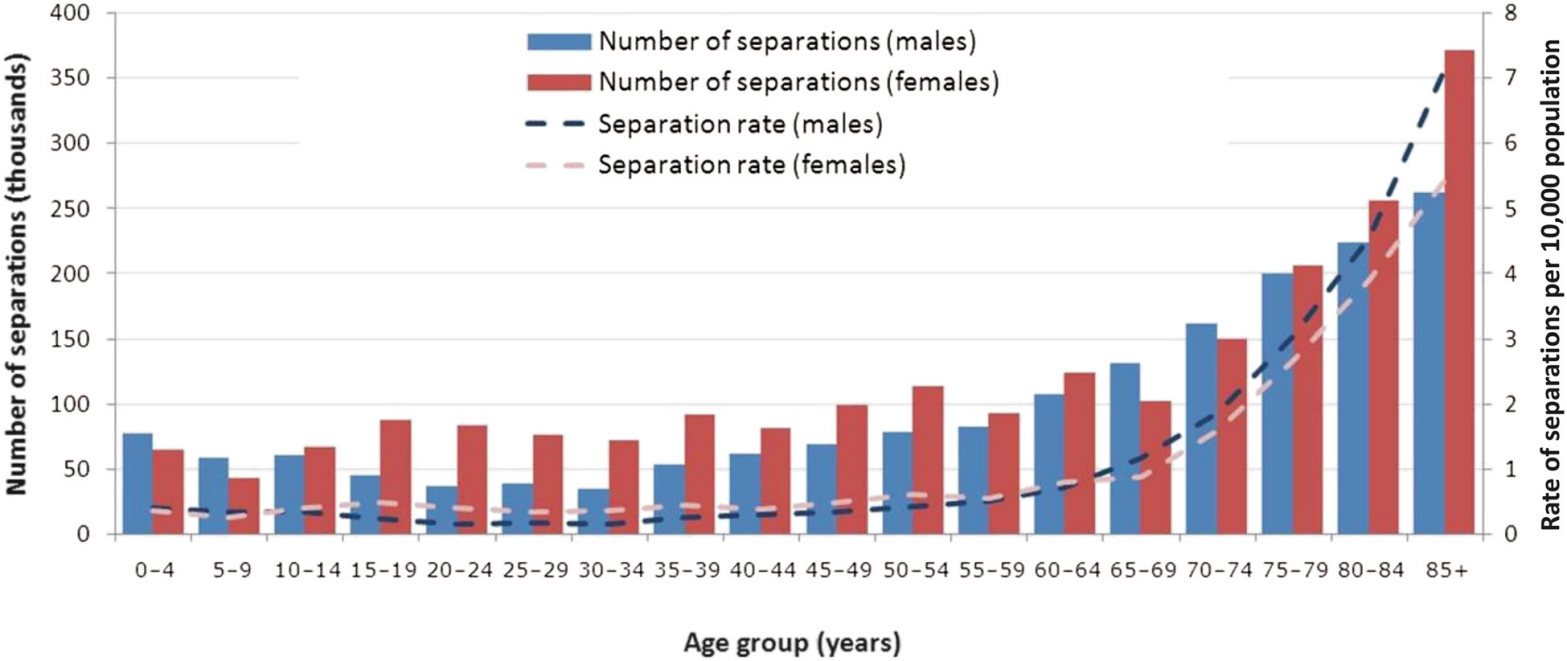
Age and sex distribution of emergency separations with a principal diagnosis constipation, Victoria, 2010–11.

**Figure 2:**
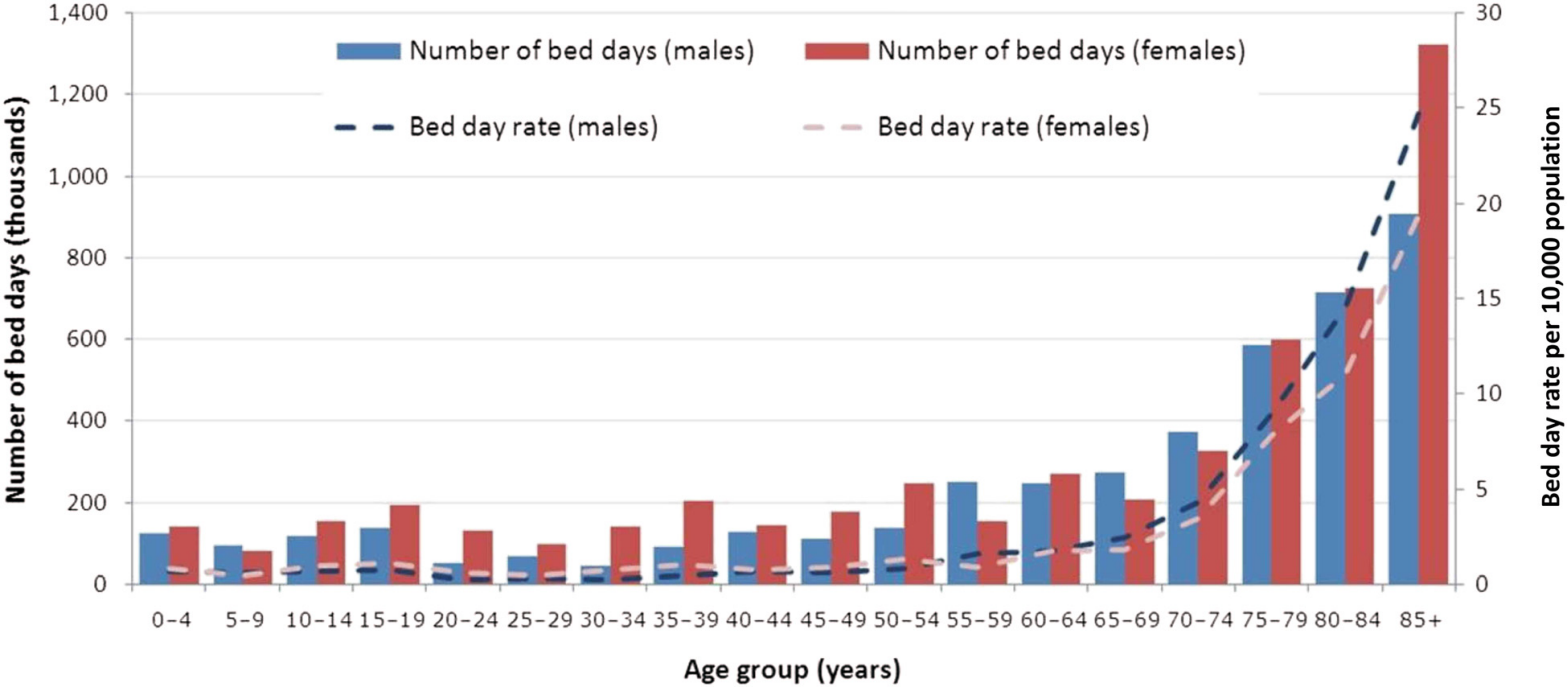
Age and sex distribution of bed days used by emergency separations with a principal diagnosis of constipation, Victoria, 2010–11.

Table 1 summarises the characteristics of the patients. Slightly more than half (55%) were female, with the median age of 65–69 years. The proportion of separations for patients in metropolitan and rural regions was similar to the proportions in the Victorian population. Most (92.2%) emergency separations for constipation were in public hospitals, 1/3 (35.2%) did not involve an overnight stay in hospital. Six per cent of separations in public hospitals were private patients.Middle-age women and older patients were more affected (Figure 1). The rates of emergency separations for constipation are greater for males from 65 years on. (lines in Figure 1). However, as there are more females than the males in older age groups, the number of separations is greater in older females than older males.The resource impact of emergency separations for constipation can be gauged by the number of bed days used and WIES funding allocated. Hospital bed days per capita reflect hospital use and are unaffected by multiple admissions or transfers between hospitals and specialties. In the financial year 2010–11, a total of 9,710 bed days were used by patients with a principal diagnosis of constipation, equating to a crude (unadjusted) emergency bed day rate of 7.1 per 10,000 of population (Figure 2).

**Table 1:** Patient and hospital-related characteristics associated with emergency separations with a principal diagnosis of constipation, Victoria, 2010–11

		**Number**	**Percent**
Gender	Male	1,790	45.0
	Female	2,188	55.0
Age group	0–14	374	9.4
	15–29	371	9.3
	30–44	398	10.0
	45–59	537	13.5
	60–74	779	19.6
	75+	1,519	38.2
Country of birth	Australia	2,600	65.4
	Overseas	1,378	37.4
Area of residence	Metropolitan	3,029	76.1
	Rural	949	23.9
IRSED 2006 quintile	1 (most disadvantaged)	872	21.9
	2	753	18.9
	3	618	15.5
	4	1,077	27.1
	5 (least disadvantaged)	658	16.5
Patient type	Public	3,301	83.0
	Private	485	12.2
	Other	192	4.8
Hospital type	Public	3,669	92.2
	Private	309	7.8
Length of stay type	Same day	1,228	35.2
	Overnight	809	31.4
	Multi-day	989	33.3
Total		3,978	100.0

An estimate for hospital inpatient costs due to constipation was based on the Australian Refined DRG to which separations were coded. Two DRGs, G70A (other digestive system diagnoses with catastrophic or severe complication and/or comorbidity, CC) and G70B (other digestive system diagnoses without catastrophic or severe CC) accounted for 17.7 and 79.2% respectively, of all emergency separations for constipation.Compared with the pattern of separations for constipation (Figure 1), the pattern of bed days is amplified in older age groups (Figure 2). Seven in 10 (72.5 per cent) of the separations in DRG G70A were for patients ≥ 65 years.

Emergency admissions for constipation in public hospitals, accounted for 2,208 WIES in inpatient activity (0.60 WIES separation). Using the distribution of WIES by hospital pay group and applying the corresponding WIES prices for different types of hospitals, $8.3 million was allocated to public hospitals to treat unplanned separations with a principal diagnosis of constipation. Applying the lowest WIES price of $3,725 to the 263 emergency separations with a principal diagnosis of constipation treated in private hospitals raises this amount a further $979,675, to $9.3 million. The WIES payment for emergency separations due to constipation does not reflect the actual costs of an inpatient admission and therefore these figures do not represent actual costs. However, they can be regarded as providing a conservative population-based estimate of the hospital-related cost of treating constipation cases.

**Figure 3:**
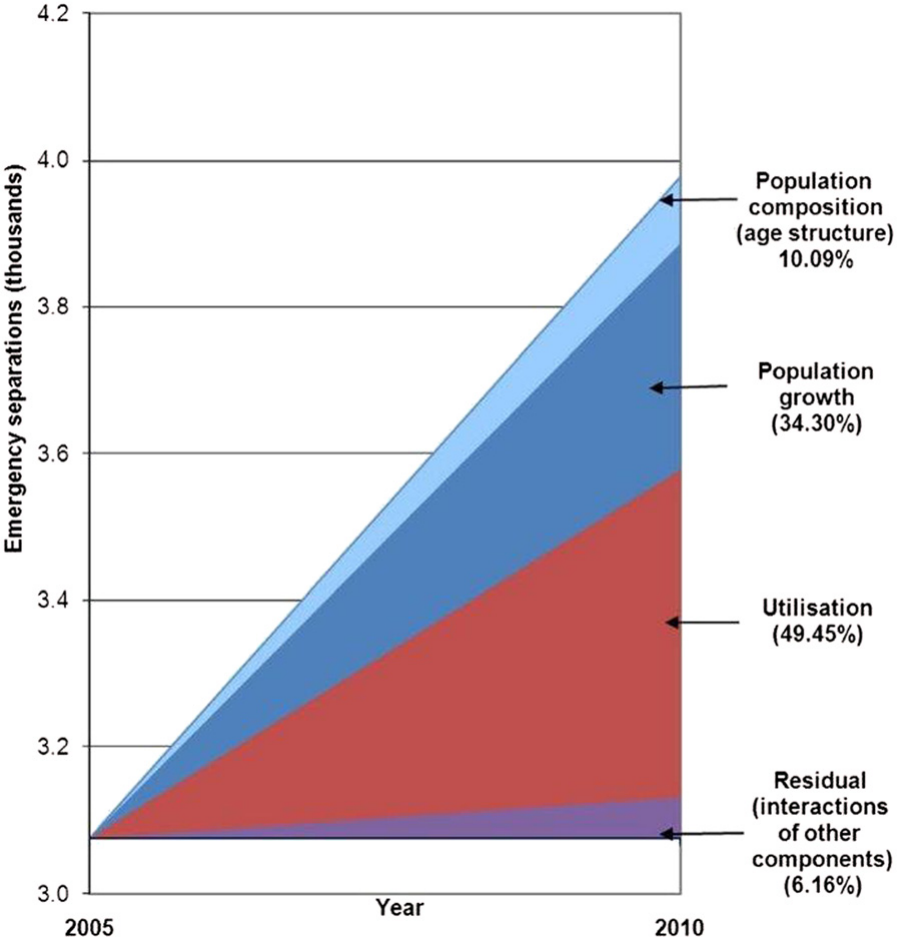
Decomposition of components of growth in emergency separations with a principal diagnosis of constipation, 2005 to 2010.

**Figure 4:**
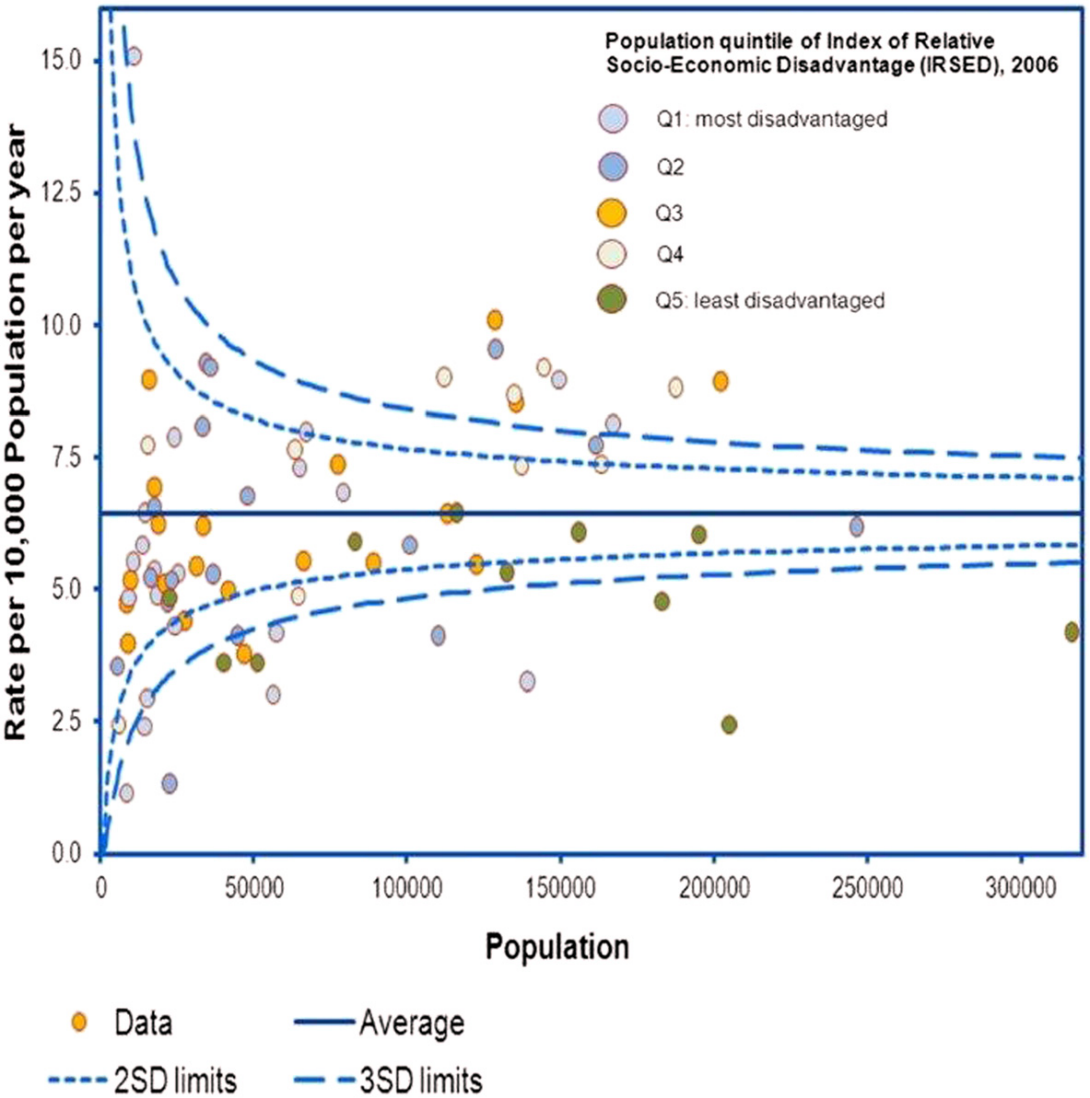
Funnel plot of adjusted separation rate, by Local Government Area, Victoria, 2009–10 and 2010–11.

Between 2005–06 and 2010–11, the number of emergency separations for constipation increased by 952 from 3,026 to 3,978 separations (31.0%). The crude (unadjusted) rate of emergency separations with principal diagnosis of constipation increased from 5.95 to 7.13/10,000 of population. Using the 2006 Victorian population as a reference population, the corresponding age-adjusted separation rates are 5.97 (5.75–6.18) and 6.98 (6.76–7.20)/10,000 population. In 2010–11, the age-adjusted separation rates were 8.4/10,000 of population for females, and 6.9/10,000 of population for males.Figure 3 shows the components that changed: demographics (population growth and ageing) and increased utilisation. This decomposition analysis shows that most (49.5%) of the increase in emergency separations with a principal diagnosis of constipation that occurred over the five years to 2010–11 was due to the increased utilisation rate in the population (Figure 3). Change in population composition (age and sex structure) accounts for 10.1% of the increase. Together, changes in population size and composition explain 44.4% of the increase.Figure 4 uses a funnel plot to show the age and sex adjusted rate of emergency separations for constipation against population size for Victoria’s 79 local government areas (LGAs). Funnel plots can be used to show the extent of the variation in rates after allowing for random variation and can be used to identify outliers. The dashed lines are the upper and lower “control limits” calculated as the 95% (2SD) and 99.8% confidence limits (3SD). To account for differences in the age and sex structure of the LGA populations, the rates were age and sex adjusted. The rates are based on indirect standardisation since the numbers of separations for LGAs with smaller populations are somewhat unstable from year to year (with the result that direct standardisation based on age and sex-specific rates may yield unreliable estimates). The rates were calculated using separations for 2009–10 and 2010–11 and are rescaled to reflect the growth that occurred over this period. The points for LGAs are colour-coded according to the quintile of socio-economic disadvantage to which each LGA is assigned (based on an area-based Index of Relative Socio-Economic Disadvantage (IRSED) that is developed from census data). When the separations for LGAs are aggregated across quintiles, the numbers are sufficient to calculate directly standardised rates. The directly standardised rates for emergency separations for constipation were 7.47 (6.98–7.96) in the most disadvantages quintile (Q1) of IRSED and 5.80 (5.38–6.21) in least disadvantaged quintile [data not shown].Emergency separations for constipation in LGAs in Victoria varied from 2.0 to 14.7 per 10,000 population with a median of 6.5 per 10,000 population. For 6 LGAs, the adjusted separation rate for constipation was above the 2SD and 3SD control limits (Figure 4). A proportion of this variation may be explained by variation in the management of constipation in primary care and other factors that were not adjusted for in the analysis. If the LGAs with higher separation rates could achieve the median rate of 6.5 separations per 10,000 population this would reduce costs.

## Discussion

The burden associated with managing emergency separations for constipation in Victoria’s hospitals is significant at nearly A$10 million/year (US $9.5 million/year). Half the increase from 2005–2011 is due to increased utilisation. This increase may be due to increased morbidity and/or an increase in the propensity of consumers to seek care from emergency departments. If a proportion of emergency separations with a principal diagnosis of constipation were avoided, there is a potential cost savings across the health care system. Although the economic burden is not great in the context of recurrent public hospital expenditure of A$9,225 million for Victoria in 2010–11, it is nonetheless not insignificant in a constrained funding environment where savings may be achievable if hospitalisation for acute constipation can be avoided either by delivering therapeutic intervention in a more cost effective setting or by preventing a condition that brings substantial suffering and decreased quality of life to the affected.

It should be recognised that use of principal diagnosis of constipation may understate the extent to which constipation is a factor that contributes to hospitalisation, especially in older patients. However, principal diagnosis is the basis casemix-based funding is assigned to episodes of care and costs are estimated. Principal diagnosis is often specified because the number of diagnosis fields reported can differ between administrative datasets, in which case the comparability can be an issue.

Many of the patients are older so more effective management of the elderly might avoid hospitalisation. Many medications that older adults take for common chronic conditions increase the risk of constipation. Constipation also occurs more commonly among people on polypharmacy therapy [14]. Cross-sectional ACSC hospitalisation data can be used to quantify the scope to avoid admissions through the provision of better ambulatory care. A study involving General Practitioners and specialists indicates a broad consensus that > 50 per cent of hospitalisations for ACSC conditions should be avoidable (6). A conventional approach is to consider the reduction in separations that could be achieved if areas with higher ACSC separation rates improved to the level of the average rate for areas with lower ACSC separation rates.

Unplanned admissions for constipation should not be regarded as inappropriate admissions or a measure of hospital performance. Emergency department care is required due to the urgent nature of conditions but some are potentially preventable/avoidable if timely and effective primary health care had been received. Treatment in Emergency Departments to provide patients with home-based disimpaction could avoid hospitalisation [15]. Barriers to primary health care may include time barriers to obtain appointments or spent in waiting rooms, travel time or distance to the practice, transportation barriers, non-availability of evening-night or weekend appointments and non-availability of primary care physicians and other health care professionals in the area.

Within public hospitals, emergency patients take up beds, staff and services from patients who have been scheduled for elective admission, particularly elective surgery. As WIES funding to public hospitals in Victoria is capped in each given financial year, emergency admissions for constipation may reduce the volume of elective admissions and increase the time that patients wait. The level of emergency separations for constipation and estimates of the activity-related resource costs involved provide an indication of the inefficiency that can stem from not accessing primary care in a timely way and/or inadequacies in the quality of the care provided in this setting at the higher end of the morbidity spectrum. Monitoring and adopting strategies to reduce ACSCs represents a method of demand management, with potential savings of 15% of $10 million, that is $1.5 million with better treatment for constipation in the primary sector.

## Conclusion

This study shows that the burden (in number of admissions, emergency bed days and overall direct costs) in managing emergency admissions for constipation in Victoria is very significant. A proportion of this burden could be alleviated by improved primary healthcare and alternative ways to achieve faecal disimpaction without emergency admission.

## Abbreviations

ACSCs: Ambulatory care sensitive conditions; DRG: Diagnosis-related group; IRSED: Index of relative socio-economic disadvantage; ICD-10-AM: International classification of diseases-10 Australian modification; VAED: Victorian admitted episodes dataset; WEIS: Weighted inlier-equivalent separation.

## Competing interests

The authors declare that they have no competing interests.

## Authors’ contributions

HA, JH, BS: study design, data collection and manuscript preparation. ZA: statistics analysis and manuscript preparation. All authors read and approved the final manuscript.

## Pre-publication history

The pre-publication history for this paper can be accessed here:

http://www.biomedcentral.com/1471-230X/14/125/prepub
